# Evolving Coronary Stent Technologies - A Glimpse Into the Future

**DOI:** 10.7759/cureus.35651

**Published:** 2023-03-01

**Authors:** Smeet Patel, Kalpen B Patel, Zeel Patel, Ashwati Konat, Ami Patel, Jinish S Doshi, Priyank Chokshi, Divya Patel, Kamal Sharma, MohmadSabir M Amdani, Darshini B Shah, Urva Dholu, Merik Patel

**Affiliations:** 1 Medicine, New York University School of Global Public Health, New York City, USA; 2 Internal Medicine, Pandit Deendayal Upadhyay Medical College, Rajkot, IND; 3 Zoology, Biomedical Technology, and Human Genetics, Gujarat University, Ahmedabad, IND; 4 Anesthesiology, Overlook Medical Center, Atlantic Health System, Summit, USA; 5 Internal Medicine, Ameer-ud-Din Medical College (AMC) Medical Education Trust (MET), Ahmedabad, IND; 6 Internal Medicine, Byramjee Jeejabhoy Medical College, Ahmedabad, IND; 7 Cardiology, Dr. Kamal Sharma Cardiology Clinic, Ahmedabad, IND; 8 Internal Medicine, Sheth Lallubhai Gordhandas Municipal General Hospital, Ahmedabad, IND; 9 Medicine, Gujarat Cancer Society (GCS) Medical College, Hospital, and Research Center, Ahmedabad, IND; 10 Internal Medicine, Gujarat Cancer Society (GCS) Medical College, Hospital, and Research Center, Ahmedabad, IND; 11 Public Health, New York University School of Global Public Health, New York City, USA

**Keywords:** in-stent restenosis, stent optimization, stent sensors, gene-eluting stents, stents, coronary artery diseases

## Abstract

One of the most widely accepted forms of treatment for coronary artery disease (CAD) is the implementation of stents into the vessel. This area of research is constantly evolving, ranging from bare-metal stents through drug-eluting stents and, more recently, approaching bioresorbable stents and polymer-free stents. This article reviews the evolution of all these devices and emphasizes how they might be further evolved to provide an optimal coronary stent and overcome unsolved challenges in stent development. We thoroughly evaluated a number of published studies in order to advance coronary stent technologies. Additionally, we looked for various literature that highlighted the inadequacies of the coronary stents that are currently available and how they might be modified to create the optimum coronary stent. Coronary stents have significantly improved clinical outcomes in interventional cardiology, but there are still a number of drawbacks, including an persisted risk of thrombosis due to endothelial injury and in-stent restenosis. Gene eluting stents (GES) and customized coronary stents with self-reporting stent sensors are appealing alternatives to existing stent approaches. Considering the adequacy of these gene eluting stents (GES), customized coronary stents produced by novel 4D printing technologies and integrated self-reporting stent sensors should be assumed for anticipating future advancements to optimal coronary stent devices; however, more interventional evidence is required to determine the future prospects of these stent innovations.

## Introduction and background

A prominent cause of death globally is coronary artery disease (CAD). In many cases, the disease is brought on by inadequate diet, hypertension, and other risk factors [1]. This disease is exacerbated by overeating, diabetes, and a lack of physical activity, all of which have contributed to the rise of a number of dangerous conditions that were unheard of just a few decades ago. Atherosclerosis, the most prevalent kind of CAD, is characterized by restricted symptoms that impede blood flow as a result of lesions in significant areas of the vasculature.

Coronary artery bypass graft (CABG), stenting, and percutaneous old balloon angioplasty are some of the invasive treatments for atherosclerosis. One of the most often used therapeutic techniques is the implantation of stents within the artery. Dexamet was the first drug-eluting stent (DES) to be introduced in the market, which was covered with Cortizone. According to the US Office of Economics, the market for implantable medical devices is anticipated to reach $400 billion in 2018. As a result, the market for stents is worth $7.98 billion, with coronary stents making up 67.3 percent of that total. Cardiovascular diseases caused 38% of the 17 million premature deaths caused by noncommunicable diseases in 2019 [2-5].

Although stent innovation was a clinical advancement, there are several disadvantages and complications related to the stents, including in-stent restenosis, a higher risk of thrombosis from endothelial damage sustained during the treatment, stent fracture, and stent thrombosis due to allergic reactions. The emergence of therapeutic drugs from the stent edges was a potential advancement in the field of cardiovascular stents [6]. The majority of in-stent restenosis (ISR), which affects around 10% of percutaneous coronary procedures, is mostly unaffected by the ongoing advancements in stent technology. Additional mechanical causes include stent expansion or fracture, and biological consequences leading to severe neointimal propagation [7]. For bare-metal stents and DES, the frequency of the composite consequence of death due to restenosis is 15.8% and 12.0%, respectively [8].

Also, DES medications should have had a preferred strategy to prevent in-stent restenosis, such as the capacity to inhibit platelet aggregation, inflammatory processes, smooth muscle cell (SMC) amplification, and migration, as well as encourage quick endothelialization and sufficient healing [9]. With the potential to improve future models, numerous additives have emerged as candidates for use in cardiovascular stent versions. Gene-eluting stents (GES) and integrated self-reporting stent sensors, both of which are under investigation, are two of the most promising.

## Review

Methods

In order to promote the development of cardiovascular stent technologies, we carefully assessed a number of published studies. Additionally, we looked for various literature that highlighted the inadequacies of the cardiovascular stents that are currently available and how they might be modified to create the optimum coronary stent, which should be safe, effective, long-lasting, easy to implant, and compatible with the patient's anatomy and medical history.

Search strategies

Using academic sites such as Google Scholar and PubMed, a comprehensive search of cardiovascular stent technologies published studies was conducted. The search approach employed terms such as "coronary artery diseases", "stents", "gene-eluting stents", "stent sensors", "stent optimization", and "in-stent restenosis" to discover targeted reporting items.

Evolution of cardiovascular stents

Drug-eluting stents (DES) have outperformed entirely bare metal stents (BMS), from restenosis frequency to delayed lumen loss, marking a turning point in the history of coronary therapeutics. DES has a metallic foundation and two additional elements: medicinal agents and a carrier of a therapeutic active ingredient. A time-programmed dosage form can strike the right balance between medication release and artery tissue permeation. Thin-strut cardiac stents, a novel metallic platform made up of various compositions, and a variety of medications, polymeric materials, and their combinations have all been successfully developed by the industry.

The three primary elements of first-generation DESs were a persistent metallic foundation, a permanent synthetic polymer coating on the framework, and a pharmaceutically active chemical integrated into the polymeric coating [10]. Sirolimus is a suitable option for stent coating because of its antibacterial, antiproliferative, and immunosuppressive properties [11]. A plant-based antiproliferative and antineoplastic drug called paclitaxel is used to treat cancer [12]. First-generation stents outperformed BMS, but they were plagued by inflammatory reactions and slow artery healing, prompting the development of second-generation stents. The development of second-generation DESs was motivated by the need to increase endothelial coverage while decreasing inflammation and enhancing delivery. The zotarolimus and everolimus stents are based on sirolimus stents. These medications function similarly to sirolimus [12]. As the first second-generation DES, the FDA approved Medtronic's Endeavour in 2004. The FDA authorized Boston Scientific Abbott's XIENCE in 2008 and Promus in 2012, respectively. Due to its enhanced performance and interventional safety, XIENCE has established a milestone in the stent business [13]. Stents constructed of persistent polymers, which do not dissolve over time and remain in place even after the polymer's medication has been eluted, are associated with problems. These stents would comprise those without polymers or those coated with biodegradable biomaterials. The tremendous technical development of the last three to four decades has had a significant impact on the advancement of stents.

Biodegradable Polymer-Coated Stents

Potential advantages of this stent include improved acute occlusion/acute recoil prevention, minimal neointimal hyperplasia, and evasion of late vessel wall inflammation. The main disadvantages are the complexity of delivering to the desired site of action and the increased risk of acute strut breakage due to low mechanical strength, the need for continuous dual antiplatelet therapy for up to two years, even with the second generation of durable polymer drug-eluting stent.

Polymer‑Free Stents

Polymers do not have anti-proliferation or healing properties. As a result, the medicine is efficiently layered on topsoil, occupying the micropores and leaving a thin film of the drug on the surface. Micron-scale pits and micropores help the drug adhere to the stent's surface and act as a storage tank for the drug.

Bioresorbable Stents (BRS)

Numerous product lines were developed for this original and distinctive idea, but none of them was able to reach the market due to issues. Long-term antiplatelet medication is no longer necessary because of this characteristic. The stent gradually disappears from the location due to bio-resorption. This kind of stent enables the channel to heal completely without being blocked [14].

Although coronary stent technology has advanced, in-stent restenosis (ISR) remains the interventional cardiologists' "demon," leading to numerous technological advancements over the past twenty years aimed at lowering its incidence. There are numerous potential therapies to combat ISR. Drug-based treatment regimens alone are not an effective strategy for lowering ISR recurrence rates; additionally, they are associated with the development of adverse events [15]. While using abciximab was thought to be efficacious in preliminary studies, this advantage was not supported by subsequent research findings [16]. Oral sirolimus administration was mentioned as a treatment option, but long-term data are insufficient. Furthermore, the increased likelihood of adverse drug reactions is yet another argument against using this treatment regimen [16-18]. ISR management with a second DES or a drug-coated balloon is superior to balloon angioplasty alone [19]. Further research into new stent materials and technology, as well as the use of brachytherapy, is essential because the best method to treat ISR has not yet been identified.

Cardiovascular stent optimization and advancement

Despite significant progress, the optimum DES device is still yet to be established. Eliminating potentially lethal consequences and in-stent restenosis should be the primary goal of the new DES. To ensure safety, effectiveness, and cost-efficiency, the perfect DES might also implement a number of emerging and upgraded components and delivery systems. Gene-eluting stents (GES) and integrated self-reporting stent sensors are two of the most promising and are currently being explored.

Gene-eluting stents (GES)

Numerous new compounds have emerged for potential use, tempting to implement sustainable cardiovascular stent configurations. Among these would be the gene-eluting stents (GES), those which are being studied. Interventional success in the cardiovascular gene therapy sector has been limited. The main cause of this is insufficient gene propagation, which has complicated and diverse effects that include systemic immune reactions to vectors, a lack of delivery mechanisms, and insufficient cell-vector interactions [3]. These obstacles can be overcome by GES by employing stents as allocation scaffolds for localized genetic exchange to the vascular system. This technique allows for prolonged elution and may even prevent a multisystem immune response. Collagen and matrix metalloproteins are prominent noncellular objectives [20]. Cardiovascular gene treatment requires a curative gene, a carrier to convey the gene, and a supplier to administer the vector. The stent coatings must be inflammatory- and thrombogenic-free and have biocompatibility [3]. After the stent is implanted, such coatings will be close to the systemic circulation, and maintaining a persistent local concentration of the medication depends on their employment as a reservoir.

DNA (Plasmid) Eluting Stents

Klugherz et al. first demonstrate the administration of green fluorescent protein (GFP) plasmid DNA into the blood vessels of a pig using merely a DNA-eluting stent for the management of cardiac illness [21]. One of the main difficulties in doing this research was the penetration of DNA in distant organs and descending coronary sites. DNA was successfully deployed to smooth muscle cells in a pig artery with great efficiency and localization using collagen-coated stents covalently coupled to an adenoviral monoclonal antibody [21]. According to Jin et al., a coronary vascular stent was created that delivered plasmid DNA to specific areas of the artery, and they detail how the plasmid DNA was stuck to the anti-DNA antibody-wrapped coronary vessel stent, it became clear that the device may be used for cardiovascular gene treatments when it was able to extract plasmid DNA with amazing accuracy and a neointimal transfection rate of about 7% [22]. Another study by Kim et al. constructed a hydroxyapatite-coated stent and used ionic characteristics in a polyplex arrangement with polyethyleneimine to deposit plasmid DNA onto the interface. This stent provided excellent-quality plasmid DNA while maintaining structural stability [23].

Vascular Endothelial Growth Factor (VEGF)/VEGF-Paclitaxel Co-eluting Stent

Yang et al. developed a PLGA nanoparticle-coated stent encapsulating VEGF and paclitaxel as a combination therapy agent for in-stent restenosis; this stent had an interior structure made of paclitaxel and maybe another core made of poly(lactic-co-glycolic acid) (PLGA) nanoparticles encapsulating VEGF [24]. VEGF genes were first released, then paclitaxel. Restenosis was significantly reduced in stents when cell multiplication was inhibited, and by four weeks after implantation, complete re-endothelialization had been achieved [24]. The VEGF and Ang-1 proteins were incorporated into the nanoparticles for targeted delivery to the specific location [25].

Interfering RNA-Eluting Stents

The control of vascular restenosis following endothelial injury is mediated by three microRNAs - 21, 145, and 221 [26]. Ji et al. found that suppressing the antisense of the miR-21 gene reduced the strength of neointimal lesions development [27]. Additionally, they found that deletion of strain similarity from chromosome 10, a usual target for miR-21 suppression, boosted the proliferation of vascular smooth muscle cells, Akt, and Bcl-2 activation, along with phosphate inhibition [27]. The carotid balloon lesion's overexpression of miR-145 increased the transcription of the VSMC biomarker while decreasing neointimal proliferation [28]. When an Akt1 siRNA-embedded stent was used, it was found to be able to control restenosis, suggesting that inhibiting the Akt1 protein, which is involved in cell expansion, could reduce cell growth. Short interfering RNA (siRNA), like microRNAs, has the potential to mute RNA [29].

Integrated self-reporting stent sensors

The same deadly outcomes as the underlying CAD can result from a vascular blockage in the region where the stent was inserted. Patient safety, healthcare costs, and the likelihood of potential catastrophic problems suddenly appearing would all be significantly impacted by the capacity to detect stent obstruction at a distance. This issue can be resolved by creating inventive and original stents with built-in sensors that keep track of circulation, differential pressure, various cell kinds, and cell growth throughout the lesion site. With sensors monitoring internal vascular conditions continuously, it is essential to correctly anticipate a patient's long-term well-being depending on the current condition. Chow et al. developed a cardiac surveillance system that can be placed beneath a cardiovascular stent. To create the apparatus, an FDA-approved stent was combined with a tiny heart pressure sensor as well as a wireless transmitter. The stent acts as an antenna in this configuration, transmitting quantified solubilization to the immediate neighborhood via a wireless telemetry transmitter [30]. By creating an application-specific integrated circuit (ASIC) for remote metering that featured a voltage regulator and radio frequency power element, Chow et al. improved their method [31]. A radio frequency-powering component was implanted on the chip in the finished device as an ideal power distribution feature. Microelectromechanical modules were crammed with ASIC for data collection [31].

Monitoring methods of stent sensors

Monitoring Approach Using Wireless Pressure Sensors

Continuous blood flow surveillance can serve as a screening, advanced detection, and alert for cardiovascular health using non-invasive technology that can be placed in the coronary arteries. The vast majority of these devices rely on passive inductor-capacitor resonant circuits with dynamic resonant frequencies referring to the biological parameter estimates to function. Reviewing the admittance of an antenna close to the implant component and connected to it via electromagnetic coupling will enable this [32,33]. In order to reduce the number of unnecessary antenna components for the wireless pressure sensor and conserve space, researchers are employing the stent as an inductive antenna to create a wireless network [34].

Computed Tomography Angiography (CTA) Monitoring Method

In-stent restenosis appears to be detected using invasive coronary angiography (ICA) because the coronary artery aperture can be seen with high clarity. The invasive nature of the ICA approach, on the other hand, is associated with a high risk of fatality and complications. Invasive coronary angiography, on the other hand, is a costly procedure that requires the services of qualified and skilled personnel [35,36]. Thick strut slices and higher-density stent metals have been shown to be more difficult to predict, and the dual-source computed tomography angiography (DSCTA) apparatus has two X-ray tubes and sensors, allowing for improved resolutions and even distortion reduction, resulting in significantly improved cardiac imaging quality [37-39].

Discussion

Because coronary stent implantation is widely regarded as a safe and well-established technique for treating CAD and because it can treat restenosis caused by both coronary balloon and catheter implantation, the development of coronary stents has piqued the interest of experts over the last decade. However, ISR, which encourages the development of DES, is a disadvantage of coronary stents. However, current treatment techniques are insufficient; thus, a new approach is required to prevent ISR. Many researchers have looked into using a DES to administer medications to suppress the multiplication of SMCs in order to defeat ISR.

Current obstacles and novel insights in cardiovascular stent technology

Many clinical applications for implantable devices have already been used to monitor or record several vital bodily functions, such as the vascular system in artery walls and strain detectors positioned on companions, as well as to check the state of the in-stent embedded in the vasculature and in-stent restenosis over the period. The scale, design, and even the advanced coatings of the in-stent architecture's most troublesome elements remain. A specific set of coating materials is required to achieve the desired thickness and consistency. Pressure sensors will improve in terms of precision, dependability, and definition. The materials utilized to create and produce the sensors, including the coatings put on them, dictate these circumstances. Reduced coating thickness enhances focus, and perylene coating, a substance with good biocompatibility and perceptual capacities, may be used in this regard [40]. Thinner struts are advocated in novel stent ideas because they will benefit more from future decreases in restenosis. To make the procedure less uncomfortable and to decrease the likelihood of in-stent restenosis, the stent that is put into the blood arteries should be as tiny as feasible. In order to prevent injury from edges, implanted devices would be made smaller using auto-zeroing techniques and artificial intelligence. Laser cutting or other fabrication methods are presently used to manufacture stents. These technologies, regrettably, could be refreshed or modified as major advancements in augmented reality, 3D printing, and deep learning [41]. Investigators at Eidgenössische Technische Hochschule (ETH) Zurich have been given access to the novel 4D digital printing techniques for the generation of cardiovascular stents with 40 times considerably smaller than those already in use [42]. Smart components, typically called stimuli-responsive materials, are used in 4D printing to generate implants using 3D printing and bioprinting methods. As a result, the 4D printed object is comparable to the 3D printed one, but the nanomaterial can transform and become dynamic in response to an input thanks to the fourth dimension, time [43]. These technical advancements pave the door for patient-specific technology that might also cater to the unique demands of each person. By creating personalized cardiovascular stents that adhere to the specified conditions and pathophysiological state of the target circulatory system, the current unresolved challenges can be reduced in this approach. This analysis emphasized the limitations and obstacles of producing implanted devices and long-term therapy. We are certain that in the future, researchers and professionals will be able to answer these unconquered challenges of optimum cardiac stents.

## Conclusions

Despite the fact that we currently have a varied spectrum of drug-eluting stents that are functioning really well, experts are exploring new ways to optimize intracoronary stent performances and minimize stent-related negative consequences. Intracoronary stent technology has advanced in phases that have revolutionized the short- and long-term consequences of percutaneous coronary interventions. Despite the fact that cardiovascular stents have added clinical benefits to interventional cardiology, stent-related complications have remained a significant concern. In order to address the constraints of the stent structure, numerous reports have been undertaken on the preferred treatment of choice. By employing stents as delivery platforms for localized gene transfer to the blood artery wall, gene-eluting stents (GES) can get beyond these restrictions, allowing for sustained elution and perhaps even averting a systemic immune reaction. A reliable method to identify in-stent restenosis and maybe wirelessly alert alterations before they become clinically evident is to use customized cardiovascular stents produced by novel 4D printing technology with integrated self-reporting stent sensors. They make it possible for interventional cardiologists to treat patients with challenging lesions. Despite significant advancements, the optimal cardiovascular stents system has yet to be established.
